# Repeated touch and needle-prick stimulation in the neonatal period increases the baseline mechanical sensitivity and postinjury hypersensitivity of adult spinal sensory neurons

**DOI:** 10.1097/j.pain.0000000000001201

**Published:** 2018-03-08

**Authors:** Nynke J. van den Hoogen, Jacob Patijn, Dick Tibboel, Bert A. Joosten, Maria Fitzgerald, Charlie H.T. Kwok

**Affiliations:** a Department of Anaesthesiology and Pain Management, Maastricht University Medical Centre+, Maastricht, the Netherlands; b Department of Translational Neuroscience, School of Mental Health and Neuroscience, Maastricht University, Maastricht, the Netherlands; c Intensive Care, Department of Paediatric Surgery, Erasmus MC-Sophia, Rotterdam, the Netherlands; d Department of Neuroscience, Physiology and Pharmacology, University College London, London, United Kingdom. Kwok is now with the Hotchkiss Brain Institute, University of Calgary, Calgary, Alberta, Canada.

**Keywords:** NICU, Neonatal sensory intervention, Tactile stimulation, Early-life pain, Postoperative

## Abstract

Noxious stimulation at critical stages of development has long-term consequences on somatosensory processing in later life, but it is not known whether this developmental plasticity is restricted to nociceptive pathways. Here, we investigate the effect of repeated neonatal noxious or innocuous hind paw stimulation on adult spinal dorsal horn cutaneous mechanical sensitivity. Neonatal Sprague-Dawley rats of both sexes received 4 unilateral left hind paw needle pricks (NPs, n = 13) or 4 tactile (cotton swab touch) stimuli, per day (TC, n = 11) for the first 7 days of life. Control pups were left undisturbed (n = 17). When adult (6-8 weeks), lumbar wide-dynamic-range neuron activity in laminae III-V was recorded using in vivo extracellular single-unit electrophysiology. Spike activity evoked by cutaneous dynamic tactile (brush), pinch and punctate (von Frey hair) stimulation, and plantar receptive field areas were recorded, at baseline and 2 and 5 days after left plantar hind paw incision. Baseline brush receptive fields, von Frey hair, and pinch sensitivity were significantly enhanced in adult NP and TC animals compared with undisturbed controls, although effects were greatest in NP rats. After incision, injury sensitivity of adult wide-dynamic-range neurons to both noxious and dynamic tactile hypersensitivity was significantly greater in NP animals compared with TC and undisturbed controls. We conclude that both repeated touch and needle-prick stimulation in the neonatal period can alter adult spinal sensory neuron sensitivity to both innocuous and noxious mechanical stimulation. Thus, spinal sensory circuits underlying touch and pain processing are shaped by a range of early-life somatosensory experiences.

## Abstract 2

Neonatal abnormal noxious and tactile stimulations facilitate the activity of spinal neurons, which leads to an altered somatosensory and pain phenotype in adulthood.

## 1. Introduction

Chronic pain affects the daily lives of 1 in 5 adults worldwide and remains undertreated.^14^ Successful therapeutic solutions for pain are hampered by the diversity in chronic pain mechanisms and individual differences in pain sensitivity.^45^ Recent research suggests that early-life exposure to abnormal sensory stimulation is a major determinant for future pain susceptibility. Neonates admitted to Neonatal Intensive Care Unit (NICU) typically receive 10 to 14 painful procedures and considerable handling each day, often without adequate preemptive analgesic.^31^ Several clinical studies have shown that such neonatal sensory interventions can lead to alterations in behavioural and neurophysiological measures of pain processing.^8,15,25,38,40,41^ Moreover, the analgesic requirement for subsequent surgery is higher in infants who have undergone surgical procedures during the neonatal period.^27^

The long-term effects of early-life injury on adult pain behaviour have been directly demonstrated in rodent laboratory models.^36^ In rodents, repeated neonatal needle pricks (NPs) lead to increased thermal and mechanical behavioural hypersensitivity in adulthood^2,20^ and hind paw incision in early life has been shown to increase reflex pain hypersensitivity to repeat surgical injury in adults.^49^ Other studies have also reported a decrease in thermal sensitivity after early-life surgeries^39^ and altered descending pain modulation after hind paw incision during the first week of life.^47^ Importantly, the immature central nervous system is highly plastic, and sensory activity in the first week of life can induce adaptive changes within the spinal dorsal horn.^36,43^ Thus, early-life NPs increase C-fibre terminal density,^20^ and early skin incision alters the spike timing dependence and local inhibitory processing in the adult spinal dorsal horn.^23,24^ These events can amplify nociceptive signalling in later life and result in hypersensitive nociceptive circuits, maintained by aberrant neuroimmune interactions.^5,35^

However, the modality selectivity of these changes is not clear. Other nonnoxious tactile sensory circuits may also be altered in these models, and indeed, similar plasticity may be induced in spinal sensory circuits after repeated neonatal tactile interventions as well as noxious procedures. Neonates in intensive care undergo repeated handling and tactile stimulation^13,17^ which may itself contribute to alterations in developing circuitry through activity-dependent mechanisms.^21^ This question can be studied most effectively at the level of dorsal horn circuits, where both innocuous and noxious sensory inputs are first integrated in the central nervous system. To date, there has been no study of the effect of repeated neonatal noxious or tactile procedures on neuronal excitability in the adult spinal dorsal horn.

This study investigates the effect of neonatal sensory interventions on the innocuous and noxious mechanical cutaneous sensitivity of adult dorsal horn neurons using in vivo electrophysiology. The neonatal interventions used were repetitive needle pricking and a matched repetitive, nonnoxious tactile stimulation using a cotton swab, compared with undisturbed controls. Because neonatal needle pricking is known to increase adult postinjury behavioural hypersensitivity,^20,44^ we also studied the postinjury tactile and nociceptive sensitivity of adult dorsal horn neurons after the same neonatal interventions.

## 2. Methods

### 2.1. Animals

All experiments were performed in accordance with the UK Animal (Scientific Procedures) Act 1986. Adult and neonatal male and female Sprague-Dawley rats were obtained from the Biological Services Unit, University College London. Animals were housed in a temperature and humidity controlled room with a 12:12-hour light/dark cycle with food and water available ad libitum. Rats were exposed to the same caging, diet, and handling throughout the experiment. Litters were weaned at postnatal day 21 (P21) and animals were housed in same sex cages of 3 to 4 littermates. Reporting is according to the Animal Research: Reporting of in vivo Experiments guidelines developed by the National Centre for Replacement, Refinement, and Reduction of Animals in Research, United Kingdom.

### 2.2. Study design

To assess the effect of repetitive sensory stimulations during the first week of life on the electrophysiological properties of spinal cord neurons in adulthood, repetitive neonatal NPs were applied to the left hind paw during the first postnatal week as described elsewhere.^19,20,44^ To control for neonatal stress and handling, age-matched littermates received tactile stimulation (with a cotton swab) on the plantar surface of the left hind paw. A separate group of animals from different litters was left undisturbed. After the first week, litters were allowed to grow into adulthood undisturbed. When adult, (postnatal weeks 6-8) a subset of the animals were anaesthetised and an incision applied to the left hind paw, which is a model of surgical/postoperative pain. In vivo extracellular single-unit recordings of spinal dorsal horn neurons were performed at baseline (before hind paw incision), and 1 to 2 and 5 to 6 days after incision. Table 1 provides an overview of the animals used in the study.

**Table 1:**
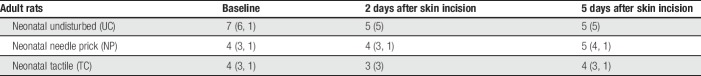
Number of animals (male and female) used in the study.

### 2.3. Neonatal procedures

New-born rat pups (n = 13) from 2 litters underwent 4 NPs per day from day of birth, postnatal day 0 (P0), to P7 as previously described.^20^ Each NP consisted of a single insertion of a sterile 25-G needle, 2 mm deep into the midplantar surface of the left hind paw. Age-matched littermates were used for the tactile stimulation (TC) group (n = 11), receiving 4 swabs with a cotton swab on the midplantar surface of the left hind paw per day, from P0 to P7. A separate group of animals from different litters was left undisturbed (UC, n = 17). Mechanical sensitivity to von Frey hair (vFh) stimulation was tested daily during the first postnatal week before (baseline), 1, 3, and 5 hours after NP application, to assess the development of hypersensitivity in the foot. Briefly, pups were lightly restrained on a bench surface, and vFhs (in grams, 0.4, 0.6, 1.0, 2.0, 4.0 [from P4 on], and 6.0 [from P6 on]) were applied 5 times to the dorsal surface of the paw, for 1 second per application. The number of positive responses (ie, withdrawal or flinching responses evoked by vFhs, guarding of the foot) was recorded and a 50% paw withdrawal threshold was calculated using sigmoidal curve fitting in GraphPad Prism 7.

### 2.4. Adult skin incision

A unilateral plantar hind paw incision was made as previously described.^7^ Briefly, at 6 to 8 weeks of age, animals were anaesthetised with isoflurane (2%-3%), an incision (1 cm long) was made along the midline through the skin and fascia of the left plantar hind paw, the plantaris muscle was then lifted and incised longitudinally. The skin was sutured with 2 mattress sutures (5-0 silk suture, Ethicon).

### 2.5. Dorsal horn electrophysiology

In vivo extracellular single-unit recordings were performed as previously described.^37^ Rats were anaesthetised with isoflurane (induction 5% in O_2_). The animals were tracheotomised; air flow and breathing rate were adjusted to animal weight with a small animal ventilator (Model 687; Harvard Apparatus, MA). Procedures were performed under constant isoflurane anaesthesia (maintenance 1.8% in O_2_, Univentor Anaesthesia Unit 400; Royem Scientific, Luton, United Kingdom). A heating blanket with feedback control was used to maintain body temperature throughout recording (Model 507220F; Harvard Apparatus). The rat was mounted on a stereotactic frame (Kopf Instruments, Tujunga, CA) and a laminectomy performed to expose the lumbar spinal cord. The vertebral column was secured with a clamp rostral to the laminectomized area, the dura and pia mater were removed, and the exposed spinal cord was covered with mineral oil.

A 6-μm tipped glass-coated carbon fibre electrode (Kation Scientific, Minneapolis, MN) was lowered through the spinal cord in 2 to 10 μm steps with a Microdrive (Scientifica Microdrive, Scientifica, Uckfield, United Kingdom). To isolate individual cells, the plantar surface of the hind paw was stroked as search stimulus for dorsal horn wide-dynamic-range (WDR) neurons in laminae IV-VI, at a depth between 400 and 800 μm. Stimulus-evoked potentials were digitalized by a Powerlab interface and recorded using Labchart software (AD Instruments Ltd, Oxford, United Kingdom). The cutaneous receptive field (RF) on the plantar skin to tactile (a camel hair brush) and noxious pinch stimulation (fine forceps) was mapped using InkScape software. Spontaneous activity in the absence of cutaneous stimulation was recorded for 1 minute. The number of spikes evoked during a single 0.5-second brush (repeated 3 times at >1 second intervals), a single 1.5-second pinch (repeated 3 times at >1 second intervals), and a single 0.5-second vFh stimulus (range: 1.202; 2.041; 3.63; 5.495; 8.511; 15.136; and 28.84 g; 3 applications to peak RF, >1 second interstimulus interval) was recorded, with a minimum 10-second interval between stimuli. A total of 313 cells were recorded from the ipsilateral and contralateral dorsal horn of 41 animals (206 ipsilateral and 107 contralateral). Outliers were removed and 298 cells (191 ipsilateral and 107 contralateral) were included in the analysis, summarized in Table 2. The depth of cells recorded and analysed per group is included in Table 3.

**Table 2:**
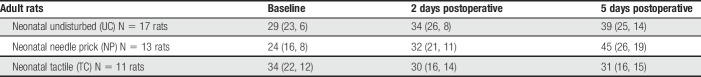
Number of wide-dynamic-range cells (ipsilateral and contralateral) recorded in each group.

**Table 3:**
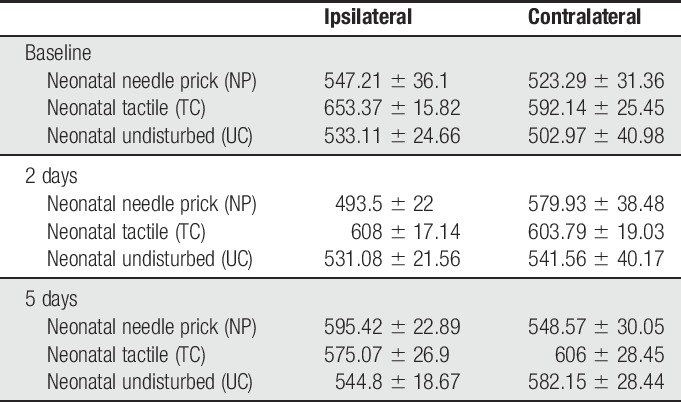
Depth of cells (μm, wide-dynamic-range neurons) recorded in the spinal dorsal horn of each group.

### 2.6. Statistical analysis

All data were plotted using GraphPad Prism 7 and presented as mean ± SEM. A *P* value < 0.05 was considered statistically significant. Statistical outliers were identified by the ROUT's method in GraphPad Prism, 15 of 313 cells recorded were excluded from analysis as outliers. Normality of data sets was checked using the D'agostino and Pearson test. As data sets were normally distributed, parametric tests were applied. Neonatal behavioural data were analysed using 2-way analysis of variance (ANOVA) with post hoc Bonferroni multiple comparison. Group differences in neuronal activity at baseline (before incision, at 6-8 weeks of age) were compared using 1-way ANOVA with post hoc Bonferroni multiple comparison. Within- and between-group differences in neuronal activity after incision were compared with the 2-way ANOVA test with post hoc Bonferroni (postincision) multiple comparison.

## 3. Results

### 3.1. Repetitive neonatal needle prick, but not tactile stimulation, transiently decreases mechanical withdrawal thresholds

**Figure 1.**
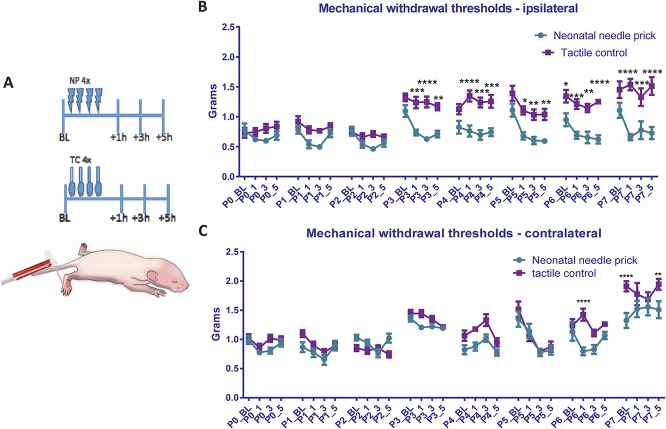
Mechanical sensitivity during the first week of life after repeated neonatal procedures. (A) The time course of the experiment. Rat pups (littermates) either received 4 tactile stimuli (cotton swab to the left hind paw, N = 9 males and 2 females) or noxious NPs (N = 10 males and 3 females). Mechanical withdrawal thresholds in the ipsilateral and contralateral paws were tested daily using calibrated von Frey filaments, at baseline (before any repeated neonatal procedures), and at 1, 3, and 5 hours after. (B and C) Changes in mechanical withdrawal thresholds after repeated neonatal procedures in the ipsilateral (B) and contralateral (C) hind paw. Mechanical withdrawal threshold increased after the third postnatal day in tactile controls. Baseline mechanical sensitivity was comparable between the 2 neonatal procedure groups. However, after NPs, ipsilateral mechanical withdrawal thresholds were significantly lower compared with tactile controls, indicating the development of mechanical allodynia from postnatal day 3 to 7. In addition, neonatal NPs led to significantly lower mechanical thresholds in the contralateral hind paw on postnatal day 6 and 7. BL, baseline measurement; NP, needle prick; P(0-7), postnatal day 0 to 7; _1, _3, and _5: measurement 1, 3, and 5 hours after NP/tactile stimulus; TC, tactile control stimulus. Data presented as mean ± SEM, *P < 0.05, **P < 0.01, ****P < 0.0001, between treatment comparisons.

Needle pricks or tactile (cotton swab touch) stimulation (TC) was applied 4 times a day to the left (ipsilateral) hind paw from postnatal day (P)0-P7 (N = 19 male, 5 female). Behavioural mechanical reflex withdrawal thresholds were tested with vFhs before and after each stimulation session at 1, 3, and 5 hours (Fig. 1A), in both the ipsilateral and contralateral hind paws. Figure 1B shows that the thresholds in the ipsilateral hind paws of TC animals are unaffected by repeated tactile stimulation, displaying the normal developmental increase in reflex withdrawal thresholds reported for naive animals.^18^ A fall in threshold relative to the TC group was observed after NPs in all time points from P3-7 in the ipsilateral hind paws (Fig. 1B; TC vs NP at P3:+1: *P* < 0.001,+3 *P* < 0.0001,+5 *P* < 0.01; P4:+1 *P* < 0.0001,+3 and +5 *P* < 0.001; P5: +1 *P* < 0.05,+3 and +5 *P* < 0.01; P6: +1 *P* < 0.001,+3 *P* < 0.01,+5 *P* < 0.0001; P7:+1 *P* < 0.0001, +3 *P* < 0.001,+5 *P* < 0.0001, 2-way ANOVA with post hoc Bonferroni). Baseline NP mechanical withdrawal thresholds recovered each day, but by the end of the week, they were significantly lower than TC baseline thresholds (P6 NP 0.95 ± 0.11 g vs TC 1.34 ± 0.11 g, *P* < 0.05, 2-way ANOVA with post hoc Bonferroni).

In addition, in the contralateral hind paws of NP animals, a fall in threshold relative to TC group was observed at 1 hour after NPs at P6, and at baseline and 5 hours after NPs at P7 (Fig. 1C; P6+1 NP 0.79 ± 0.25 g vs TC 1.4 ± 0.38 g, *P* < 0.0001; P7_BS NP 1.32 ± 0.46 g vs 1.9 ± 0.32 g, *P* < 0.0001; P7+5 NP 1.51 ± 0.53 g vs 1.95 ± 0.34 g, *P* < 0.01, 2-way ANOVA with post hoc Bonferroni).

### 3.2. Baseline sensitivity to cutaneous brush and noxious pinch stimulation is increased in adult dorsal horn neurons after early-life touch and needle prick

**Figure 2.**
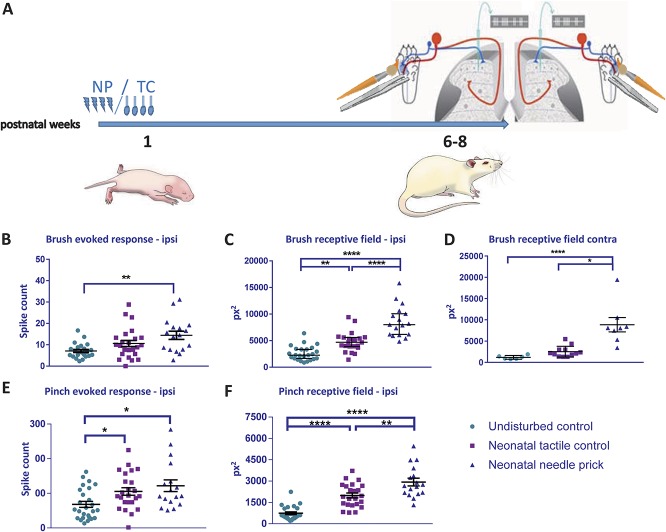
Baseline sensitivity to cutaneous brush and noxious pinch stimulation in adult dorsal horn neurons after neonatal repeated procedures. (A) Time course of the experiment. Pups received repeated NP or TC neonatal stimuli as shown in Figure 1. A different group of pups were left undisturbed (UC). At 6 to 8 weeks of age, single-unit extracellular recordings were performed in the ipsilateral (B–E) and contralateral (F) spinal dorsal horn. Cutaneous brush– and pinch-evoked spike activity and receptive field areas were recorded in wide-dynamic-range neurons. (B) Brush-evoked spike activity was significantly greater in NP animals compared with UC. (C) Brush receptive field areas were significantly larger in NP and TC animals compared with UC, and significantly larger in NP compared with TC. (D) Pinch-evoked firing activity was significantly greater in NP compared with UC. (E) Repeated neonatal procedures significantly increased pinch receptive fields in adulthood, and this effect was greatest in NP animals. (F) Brush receptive fields were largest in the contralateral dorsal horn of NP animals. NP, needle prick; px^2^, pixel^2^; TC, tactile control stimulus. Data presented as mean ± SEM. *P < 0.05, **P < 0.01, ****P < 0.001, ****P < 0.0001, between treatment comparisons.

The rats were left to grow up to 6 to 8 weeks old and the properties of adult dorsal horn WDR sensory neurons were investigated using in vivo extracellular single-unit electrophysiology (Fig. 2A). Neurons with RFs on the plantar surface of the ipsilateral and contralateral hind paw were recorded from the L4/5 dorsal horn in NP, TC, and naive (neonatally undisturbed) adult rats. Although ipsilateral measurements provide information on sensory processing in the neonatally stimulated dermatome, contralateral measurements indicate global changes in cutaneous mechanosensitivity. Baseline sensitivity of WDR cells to dynamic tactile (brush) stimulation of the plantar skin, measured as mean spikes per stimulus and mean RF area, is shown in Figures 2B–D. Baseline sensitivity to noxious mechanical (pinch) stimulation, using the same measures, is shown in Figures 2E and F.

Mean spikes per stimulus to brush was significantly greater in NP animals compared with naive animals at baseline (Fig. 2B; F(2, 61) = 6.671, *P* < 0.01, naive vs NP: 7.1 ± 0.71 vs 14.45 ± 1.92; *P* < 0.01, 1-way ANOVA with post hoc Bonferroni), but not in TC animals. Contralateral cells were unaffected. Ipsilateral brush RF area at baseline was significantly larger in both TC and NP animals compared with naive controls (Fig. 2C; F(2, 60) = 41.71, *P* < 0.0001, 1-way ANOVA), with mean NP RFs being significantly larger than TC (naive vs TC: 2614 ± 275.9 vs 4767 ± 394.7, *P* < 0.01; naive vs NP 2614 ± 275.9 vs 8590 ± 724.2, *P* < 0.0001; TC vs NP 4767 ± 394.7 vs 8590 ± 724.2, *P* < 0.0001; 1-way ANOVA with post hoc Bonferroni). The mean brush RF area in the contralateral paw of NP animals was also significantly greater compared with naive and TC animals (Fig. 2D; naive vs NP 1175 ± 177.6 vs 8837 ± 1690, *P* < 0.0001; TC vs NP 2511 ± 369.6 vs 8837 ± 1690, *P* < 0.05; 1-way ANOVA with post hoc Bonferroni).

Mean spikes per pinch stimulus were also significantly greater in TC and NP animals than in naive animals at baseline (Fig. 2E; F(2, 63) = 4.96, *P* < 0.01; naive vs TC 68.08 ± 8.72 vs 105.4 ± 10.73, *P* < 0.05; naive vs NP 68.08 ± 8.72 vs 111.8 ± 14.2, *P* < 0.05; 1-way ANOVA with post hoc Bonferroni). In addition, mean ipsilateral pinch RF area was significantly larger in both TC and NP animals compared with naive controls and was largest in NP animals (Fig. 2F; F(2, 61) = 38.23, *P* < 0.0001, naive vs TC 742.1 ± 97.6 vs 1991 ± 170.9, *P* < 0.0001; naive vs NP 742.1 ± 97.6 vs 2933 ± 267.5, *P* < 0.0001; TC vs NP 1992 ± 170.9 vs 2933 ± 267.5, *P* < 0.01; 1-way ANOVA with post hoc Bonferroni).

### 3.3. Postinjury sensitivity to cutaneous dynamic tactile and noxious pinch stimulation is increased in adult dorsal horn neurons after early-life touch and needle prick

**Figure 3.**
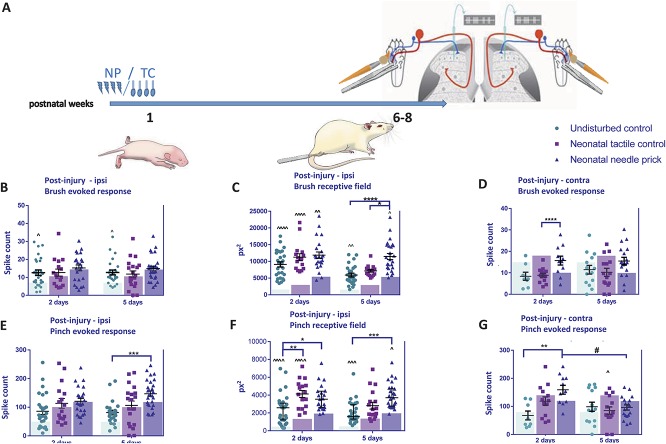
Postinjury sensitivity to cutaneous brush and noxious pinch stimulation in adult dorsal horn neurons after neonatal repeated procedures. (A) Time course of the experiment. Pups received neonatal stimulation or were left undisturbed. At 6 to 8 weeks of age, animals received an incision to the left hind paw on the plantar surface. Single-unit extracellular recordings were performed in the ipsilateral (B–E) or contralateral (F and G) spinal dorsal horn 2 and 5 days later. Cutaneous brush– and pinch-evoked spike activity and receptive field areas of wide-dynamic-range neurons were recorded. (B) No changes were observed in brush responses between groups, but brush-evoked firing was significant higher in naive animals, at both 2 and 5 days after incision compared with baseline (before incision, shown as transparent coloured bars). (C) After incision, brush receptive sizes were significantly larger in all animals compared with baseline. Between-group differences were also observed. At 5 days after incision, brush receptive fields were larger in TC and NP animals compared with naive. (D) Pinch-evoked firing activity after incision increased in NP animals compared with UC at 5 days after incision. (E) Pinch receptive field postincision was also significantly larger in all animals after incision compared with baseline. At 2 days, pinch receptive fields were significantly larger in TC and NP animals compared with naive. At 5 days, pinch receptive fields were larger in NP animals compared with naive. (F) Significant increases in postincision brush responses were also observed for NP animals in the contralateral dorsal horn after 2 days. (G) Contralateral pinch-evoked firing activity after incision was significantly enhanced in NP animals at day 2 postincision. NP, needle prick; px^2^, pixel^2^; TC, tactile control stimulus; UC, undisturbed. Data presented as mean ± SEM, transparent coloured bars represent the baseline responses of each group. ^*P* < 0.05, ^^*P* < 0.01, ^^^*P* < 0.001, ^^^^*P* < 0.0001, within treatment comparison, compared with baseline. *P < 0.05, ***P < 0.001, ****P < 0.0001, between treatment comparisons. #*P* < 0.05, between postincision day comparison.

The results above showed significant increases in adult dynamic tactile and noxious mechanical sensitivity in NP and TC dorsal horn cells measured at baseline. We next asked whether postinjury sensitivity of WDR neurons is also changed in adult NP, TC animals, using plantar incision, a model of postoperative hypersensitivity (Fig. 3A).

The mean number of spikes evoked by brush stimulation changed relative to baseline at 2 and 5 days after incision in the naive, but not NP and TC groups (Fig. 3B; effect of procedure F(2, 187) = 7.07, *P* < 0.01; effect of day F(2, 187) = 3.21, *P* < 0.05; UC BL: 7.1 ± 3.4; 2 days: 12.6 ± 8.0; 5 days: 12.7 ± 6.8; BL vs 2 days *P* < 0.05; BL vs 5 days *P* < 0.05, 2-way ANOVA with post hoc Bonferroni). However, significant increases in mean brush RF area were observed after incision compared with baseline (Fig. 3C) in all treatment groups. In naive animals, brush RF increased after incision; this increase was stronger at 2 days compared with 5 days after incision (Fig. 3C; effect of procedure F(2,127) = 14.44, *P* < 0.0001; effect of day F(2,127) = 13.69, *P* < 0.001; BL: 2614 ± 275.9; 2 days: 9095 ± 779; 5 days: 5915 ± 563; BL vs 2 days *P* < 0.0001; BL vs 5 days *P* < 0.001; 2-way ANOVA with post hoc Bonferroni). In TC animals, brush RF increased at both 2 (Fig. 3C; BL: 4767 ± 394.7; 2 days: 11,255 ± 1054; BL vs 2 days *P* < 0.0001; 2-way ANOVA with post hoc Bonferroni). Similarly, in NP animals, RF area increased at 2 and 5 days after incision (Fig. 3C; BL: 8590 ± 724.2; 2 days: 11,854 ± 976; 5 days: 11,465.85 ± 909.91; BL vs 2 days *P* < 0.05; BL vs 5 days *P* < 0.05, 2-way ANOVA with post hoc Bonferroni).

Differences in postincision brush RF sizes across all treatment groups were also tested. At 2 days after incision, no significant differences were observed between naive, TC, and NP animals. At 5 days after incision, brush RF was largest in NP animals compared with both naive and TC animals (Fig. 3C; NP vs naive: 11,465.85 ± 909.91 vs 5915 ± 563, *P* < 0.0001, NP vs TC: 11,465.85 ± 909.91 vs 7161.08 ± 400.32, *P* < 0.05; 2-way ANOVA with post hoc Bonferroni).

Significant increases in brush-evoked activity were also observed in the contralateral dorsal horn after 5 days, where NP animals showed an increase in mean number of spikes per stimulus 2 days after incision compared with TC animals (Fig. 3D, TC vs NP 8.89 ± 0.96 vs 15.68 ± 0.14, *P* = 0.037; 2-way ANOVA with post hoc Bonferroni).

We next investigated the sensitivity of WDR neurons to pinch stimulation in NP, TC, and naive animals after plantar incision in the left hind paw. Neonatal repeated procedures significantly impacted on pinch-evoked activity of dorsal horn neurons after plantar incision (Fig. 3E; effect of procedure F(2,130) = 10.18, *P* < 0.0001, 2-way ANOVA). In all 3 groups, the number of spikes evoked by noxious pinch stimulation on the RF was not significantly altered compared with baseline after incision. Pinch-evoked firing was significantly higher in NP animals 5 days after incision compared with naive animals (Fig. 3E; 5 days naive vs NP 81.38 ± 8.54 vs 147.2 ± 9.5, *P* < 0.001; 2-way ANOVA with post hoc Bonferroni).

The mean pinch RF was larger after incision compared with baseline (Fig. 3F) in all treatment groups. In naive animals, RFs were significantly larger at both 2 and 5 days after incision (BL: 742.1 ± 97.6; 2 days: 2433 ± 342; 5 days 2130 ± 248; BL vs 2 days *P* < 0.0001; BL vs 5 days *P* < 0.001; 2-way ANOVA with post hoc Bonferroni). In TC animals, pinch RFs were significantly larger at 2 d (BL: 1991 ± 170.9; 2 days: 4128 ± 397; *P* < 0.0001; 2-way ANOVA with post hoc Bonferroni) and in NP animals, at 5 days after incision (BL: 2933 ± 267.5; 5 days: 3958.78 ± 287.55; *P* < 0.05; 2-way ANOVA with post hoc Bonferroni). We also compared pinch RF sizes between treatment groups at 2 and 5 days after incision. Needle-prick animals had enlarged pinch RFs compared with naive at 2 days (NP vs naive: 4320 ± 521 vs 2433 ± 342, *P* < 0.05; 2-way ANOVA with post hoc Bonferroni) and 5 days after incision (NP vs naive: 4308 ± 445 vs 2130 ± 248, *P* < 0.001; 2-way ANOVA with post hoc Bonferroni). In addition, at 2 days after incision, RFs in TC animals were larger compared with naive (naive vs TC: 2433 ± 342 vs 4127.53 ± 396.98, *P* = 0.01; 2-way ANOVA with post hoc Bonferroni).

In addition, contralateral pinch-evoked firing after incision was significantly enhanced in NP animals 2 days after incision compared with naive animals, and decreased to baseline values 5 days after incision (Fig. 3G, 2 days naive vs NP: 83.17 ± 21.9 vs 127.6 ± 44.63, *P* = 0.01; 2 days NP vs 5 days NP: 127.6 ± 44.63 vs 96.06 ± 39.43, *P* < 0.05; 2-way ANOVA with post hoc Bonferroni).

### 3.4. Sensitivity to punctate mechanical stimulation is increased in adult dorsal horn neurons after early-life touch and needle prick

**Figure 4.**
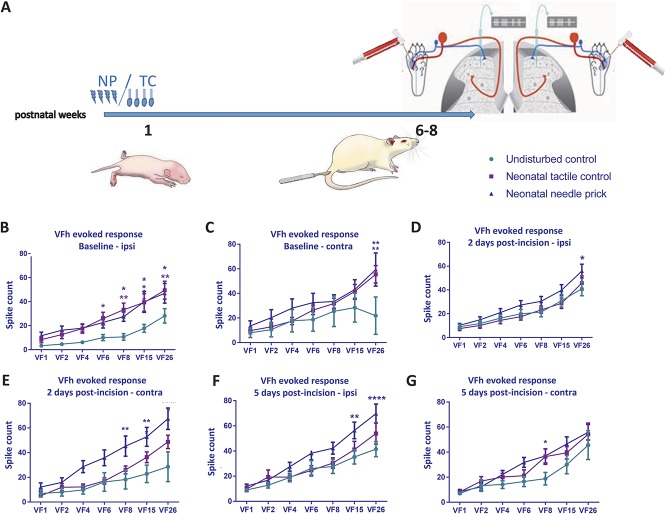
Baseline and postinjury sensitivity to cutaneous punctate mechanical stimulation in adult dorsal horn neurons after neonatal repeated procedures. (A) Time course of the experiment. Responses in WDR neurons were recorded after graded von Frey hair (vFh) stimulation at baseline and postinjury in the ipsilateral (left panel) and contralateral (right panel) dorsal horn. Data were plotted as stimulus response curves (B) Baseline vFh sensitivity ipsilateral to injury was increased in NP animals and TC animals compared with UC. (C) Needle-prick animals displayed significantly increased vFh-evoked firing at 26 g in contralateral WDRs. (D) Two days after incision injury, ipsilateral stimulus response curves indicated slight hypersensitivity in NP compared with TC and UC at the highest vFh strength. (E) Two days after incision, NP animals were hypersensitive compared with TC and UC littermates in contralateral WDRs. (F) Five days after incision, NP animals were significantly more sensitive in ipsilateral WDRs than TC and UC. Changes were also observed in the contralateral dorsal horn. (G) Five days after incision, vFh-evoked contralateral neuronal firing was highest in the NP group. NP, needle prick; TC, tactile control stimulus; UC, undisturbed; vFh/VF, von Frey filament; WDR, wide-dynamic range. In all figures: data presented as mean ± SEM, *P < 0.05, **P < 0.01, ***P < 0.001, ****P < 0.0001, between treatment comparisons. Asterisks are colour coded; dark blue asterisks represent between neonatal NP and undisturbed control comparisons; purple asterisks represent between neonatal tactile control and undisturbed control comparisons.

Finally, we measured the sensitivity of dorsal horn WDR cells to graded mechanical punctate stimulation on the plantar skin with vFhs, in naive, TC, and NP animals (Fig. 4A). The average response of 3 × 0.5 seconds applications of each vFh was used to plot a stimulus–response curve for each neuron at baseline, 2, and 5 days after incision (Figs. 4B–G).

Baseline firing increased with vFh stimulus intensity in all groups, both ipsilateral and contralateral to treatment side (Figs. 4B–G, ipsilateral overall vFh effect: F(6,366) = 46.71, *P* < 0.0001; contralateral overall vFh effect: F(6,161) = 9.244, *P* < 0.0001; 2-way ANOVA with post hoc Bonferroni). However, animals that received repeated neonatal procedures (TC and NP) were more sensitive to the higher strengths of vFh when applied to the ipsilateral RF (6-26 g) compared with naive (Figs. 4B–C; ipsilateral overall treatment effect: F(2,61) = 6.402, *P* < 0.01; contralateral overall treatment effect: F(2,161) = 6.081, *P* < 0.01; 2-way ANOVA with post hoc Bonferroni).

The postinjury sensitivity to vFhs was also tested after skin incision. After incision, vFh-evoked firing relative to baseline in naive animals increased in response to 15 and 26 g vFh at 2 days after incision (Figs. 4B, D, and F; VF 15 BL vs 2 days: 18.65 ± 16.72 vs 30.9 ± 27.9, *P* < 0.05; VF26 BL vs 2 days: 28.14 ± 30.8 vs 40.99 ± 30.5, *P* = 0.017; 2-way ANOVA with post hoc Bonferroni), and to 15 and 26 g at 5 days after incision in NP animals (VF15 BL vs 5 days 18.65 ± 16.72 vs 35.33 ± 28.6, *P* < 0.01; VF26 BL vs 5 days: 28.14 ± 30.8 vs 69.75 ± 39.6, *P* < 0.0001; 2-way ANOVA with post hoc Bonferroni). In addition, in NP animals, vFh-evoked firing in the contralateral dorsal horn in response to 8, 15, and 26 g vFh increased 2 days after incision, and normalized compared with naive and TC animals 5 days after incision (Figs. 4C, E, and G; 2 days naive vs NP VF8: 18.21 ± 21.78 vs 45.3 ± 27.18, *P* = 0.0036; VF15: 22.83 ± 20 vs 52.82 ± 25.47, *P* = 0.0010; VF26: 28.58 ± 34.2 vs 67.52 ± 28.85, *P* < 0.0001; 2 days TC vs NP VF8: 26 ± 12.13 vs 45.3 ± 27.18, *P* = 0.0225; VF26: 48.67 ± 20.43 vs 67.52 ± 28.85, *P* = 0.027; 2-way ANOVA with post hoc Bonferroni).

## 4. Discussion

The aim of this study was to measure the effect of repeated neonatal sensory (painful and tactile) interventions on the sensitivity of spinal dorsal horn neurons in adulthood. We show, for the first time, that baseline innocuous and noxious mechanical sensitivity of ipsilateral spinal dorsal horn neurons is altered by both repeated tactile and NP in early life. Significant increases in neuronal sensitivity were also observed in the contralateral dorsal horn, suggesting widespread changes in spinal sensory processing. Neuronal responses to mechanical stimulation in adulthood is further enhanced in the presence of surgical skin injury. Hence, sensitivity of spinal dorsal horn neurons to a range of mechanical stimulus modalities is enhanced by repetitive neonatal sensory intervention. Although repeated noxious interventions have the greatest effects, repeated tactile interventions are also significant.

### 4.1. Sensitisation of tactile and nociceptive spinal circuits as a result of abnormal sensory stimulation during the early postnatal period

The maturation of the central nervous system is activity dependent, functional touch and pain modulation requires balance of both excitatory and inhibitory activity within the sensory network,^4,33,34^ and appropriate sensory signals from the periphery.

Electromyographic studies have shown that maturation of nociceptive withdrawal reflexes takes place over the first 3 postnatal weeks in the rat.^46^ Blockade of low-threshold afferent sensory inputs from the tail with local anaesthesia during the neonatal period inhibited refinement of tail-flick reflex, but preservation of tactile input by replacing anaesthesia with short-acting analgesic can induce adaptation in the withdrawal response.^46^ Other types of neonatal sensory deprivation, such as maternal separation, are routinely used to model early-life stress.^10,50^ Separation of male rat pups from the dam for 3 hours daily between postnatal days 2 to 14 increased visceromotor responses to phasic colonic distension at 2 months of age.^10^ In addition, repetitive perioperative administration of sciatic block at the time of neonatal surgery in rats inhibited the subsequent hyperalgesic responses on reinjury at the same dermatome.^49^ Thus, aberrant primary afferent activity may induce long-lasting hypersensitivity within central sensory circuits, which in turn alter the maturation of nociceptive processing.

Our results showed that repetitive NP in rats, a model for noxious experiences in infants admitted to neonatal intensive care unit (NICU), increased the duration of postsurgical pain sensitivity in adults.^44^ This is in line with other models of early-life injury which revealed long-lasting sensory behavioural changes.^29,49^ Importantly, in this study, we focussed on activity within the spinal dorsal horn. The spinal cord dorsal horn is the hub of pain processing and neuronal hyperexcitability within this region is a fundamental mechanism for pain sensitisation.^4^ By recording directly from dorsal horn neurons, rather than behavioural or reflex measures, we causally established that abnormal early-life sensory interventions directly alter baseline stimulus-evoked spike activity, and cutaneous RF areas of wide-dynamic neurons in the adult spinal cord. Importantly, these changes are not restricted to nociceptive, but also brush sensitivity. This highlights an advantage of in vivo dorsal horn electrophysiology where both innocuous and noxious processing can be examined in detail.

### 4.2. Shaping adult pain phenotype by neonatal noxious experience

In addition to baseline changes, we show that repetitive NPs during the first week of life in the rat strongly enhanced neuronal responses to both tactile and noxious stimulation after plantar hind paw incision (surgical pain) in the same dermatome during adulthood.^29,49^ Specifically, animals that received neonatal sensory interventions did not exhibit an increase in evoked firing after incision during adulthood per se, rather an expansion in RFs. This could be due to heightened basal activity of spinal neurons, which were not able to increase further in the presence of an injury. Expansion of RF areas suggests an increase in recruitment of sensory afferent terminals, perhaps by disinhibition,^3^ ultimately driving pain sensitization by spatial summation.^16,52^

Plantar hind paw incision during adulthood facilitated neuronal activity in response to mechanical stimulation in naive animals. This effect was only observed in the ipsilateral hind paw mirroring the time course of surgical pain behaviours,^7,51^ suggesting that a single, acute experience of surgical pain during adulthood may not be sufficient to drive global sensitization.

One limitation of this study is that it was not powered to detect sex differences. Male, but not female rats subjected to repeated needle pricking during the first week of life develop mechanical hypersensitivity to intraplantar injection of complete Freund adjuvant during adulthood.^19^ Similarly, intraplantar incision in P3 rats leads to mechanical, heat, and cold hypersensitivity in the ipsilateral hind paws at 4 to 6 weeks of age.^9^ Nonetheless, our findings and others converge onto an important conclusion: early-life sensory alterations affect maturation of sensory processing, which leads to an altered pain phenotype in adulthood. One further important point relates to the reported baseline global hyposensitivity in behavioural studies after early-life injury.^28,48,49^ Here, we examined such global effects by recording from dorsal horn neurons contralateral to the treatment. We found that baseline brush activity in the contralateral dorsal horn was increased by neonatal repetitive NPs and that contralateral dorsal horn neuron brush and pinch responses were enhanced after plantar hind paw incision in adulthood. This widespread hypersensitivity agrees with previous single-unit electrophysiology studies and mapping of individual neuronal RF sizes, where neonatal surgery increased neuronal RF sizes at 6 weeks of age^42^ and highlights the importance of not relying reflex behaviour alone when studying sensory and pain processing.

### 4.3. Early-life tactile stimulation also affects adult pain processing

The maturation of sensory processing may be affected by several factors. In our study, we controlled for general handling or maternal separation stress by testing our NP animals alongside TC animals. Littermates were allocated to both the TC and NP group reducing any variation caused by rearing. Because neuronal hypersensitivity was persistently the strongest in NP animals, regardless of the stimulation modalities applied, pain hypersensitivity is selective to early-life pain experiences. However, the data also clearly show that repeated tactile stimulation is able to alter dorsal horn touch and pain processing. Infant rats are highly sensitive to tactile stimulation^21^ and this stimulation may recruit many of the same processes, to a lesser extent than noxious stimulation. In clinical studies, changes in skin conductance and behavioural arousal have been reported to be the same for noxious (heel prick) or tactile (routine nursery handling) stimulations,^17^ and a significant increase in behavioural and physiological “pain” scores has been reported on tactile stimulation compared with baseline.^13^ Considerable research has been undertaken in the developmental plasticity of the tactile system,^11^ including the effect of maternal licking and grooming during the first week of life.^26^ Thus, there is an increasing understanding of the dorsal horn circuitry underlying touch.^1^ Altogether, our findings and others underline the importance of future research in the effects of repeated handling of human neonates on their adult touch and pain processing.

### 4.4. Cellular and molecular mechanisms underlying neonatal somatosensory priming of nociceptive circuits

Neonatal injuries were shown to alter the pattern and density of afferent fibre terminations in the spinal dorsal horn.^12,20,32^ The retraction of low-threshold A fibres from the superficial dorsal horn during the first 3 postnatal week of the rat is important for the refinement of withdrawal reflexes and maturation of sensory thresholds.^12,30^ If this process is disrupted, it leads to a hypersensitive phenotype in later life.^6^ Moreover, the higher expression of calcitonin gene-related peptide found in adult animals after neonatal repetitive procedural pain suggests an increase in nociceptive afferent sprouting.^20^ Importantly, early-life injuries were shown to cause substantial alterations in spike timing-dependent plasticity^23^ and a failure of glycinergic inhibition,^24^ both of which enhanced dorsal horn neuron sensitivity. These neonatal interventions also occur at a period of tonic descending excitation of spinal dorsal horn neurons from brainstem control centres,^37^ which may be permanently altered by these events,^47^ for instance by increasing the opioid tone in the periaqueductal gray.^22^

Substantial evidence supports a role of microglia in sensory development and plasticity. An increase in microglial reactivity accompanied enhanced pain behaviours to reinjuries in animals that had early-life noxious experiences.^5^ Greater phosphorylation of the signalling enzyme p-38 mitogen-activated protein kinase (p38-MAPK) in microglia was reported in adult rats receiving plantar hind paw incision, and blockade of p-38 MAPK reduced pain behaviours in incised adults with previous neonatal injury.^35^ As microglia are also important for maintenance of synaptic functions, the significance of microglia in mediating changes of neuronal outputs during early development warrants further investigation.

### 4.5. Implication of findings and possible interventions

In conclusion, our study used clinically relevant models of early-life sensory interventions and showed that neonatal abnormal noxious and tactile stimulations persistently facilitate the activity of spinal neurons in both baseline and postsurgical conditions. Our findings and others converge onto an important conclusion: early-life sensory alterations affect maturation of sensory processing, which in turn leads to an altered somatosensory and pain phenotype in adulthood.

## Conflict of interest statement

The authors have no conflict of interest to declare.

This work was supported by the Pain Knowledge Centre Maastricht and Pain Knowledge Centre Rotterdam (B.A.J., D.T., and J.P.) and the Medical Research Council UK, G0901269 (M.F.).
